# Supplementary material for: Functional Characterization of POFUT1 Variants Associated with Colorectal Cancer
Source: Cancers (Basel). 2020 May 31;12(6):1430. doi: 10.3390/cancers12061430 (PMC7352195; doi:10.3390/cancers12061430)
Supplement: Supplementary file 1 [file cancers-12-01430-s001.pdf]

Supplementary Materials

Functional characterization of POFUT1 variants associated with colorectal cancer

Marlène Deschuyter, Florian Pennarubia, Emilie Pinault, Sébastien Legardinier and Abderrahman Maftah

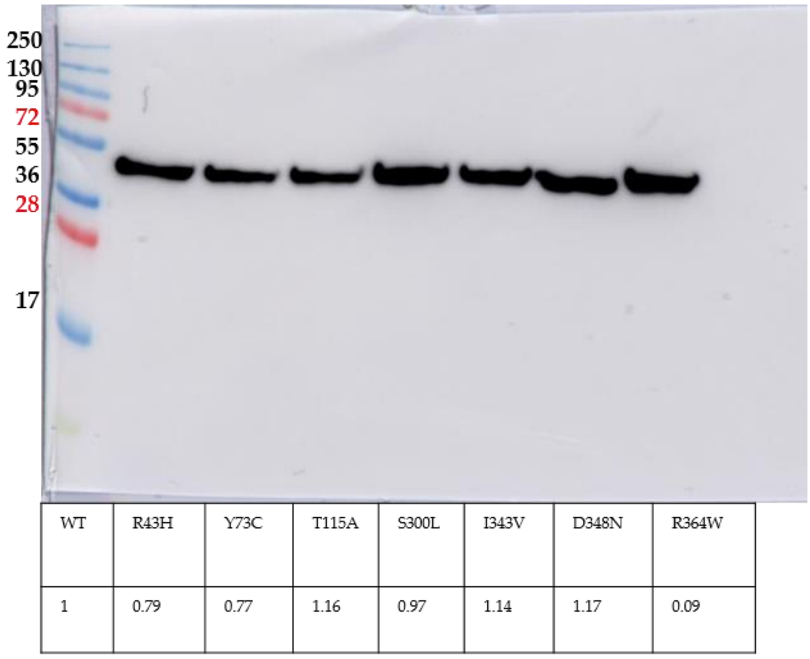

WT	R43H	Y73C	T115A	S300L	I343V	D348N	R364W
1	0.79	0.77	1.16	0.97	1.14	1.17	0.09

A

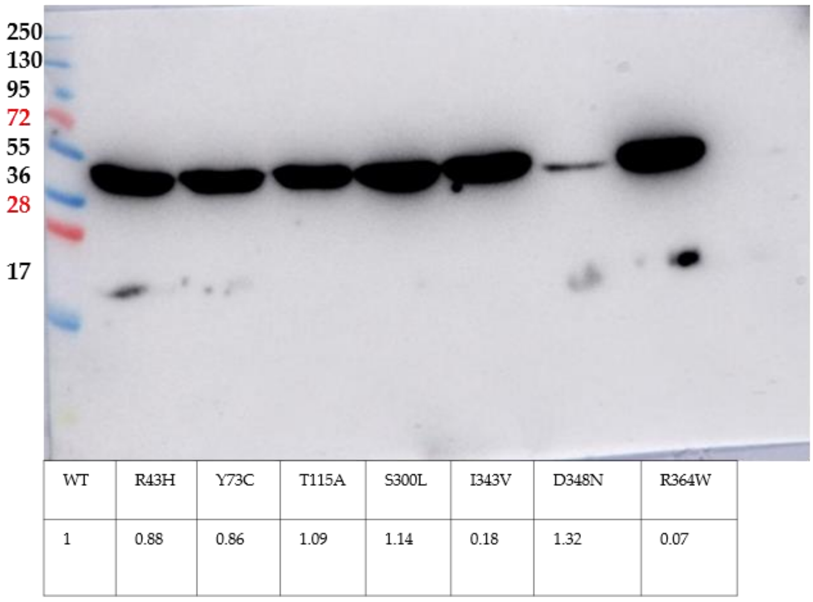

WT	R43H	Y73C	T115A	S300L	I343V	D348N	R364W
1	0.88	0.86	1.09	1.14	0.18	1.32	0.07

B

Figure S1. Uncropped blots of Figure 2. Western blot analysis, using anti-POFUT1 antibody, of recombinant POFUT1 variants, secreted by stable CHO cell lines with (**A**) 0% fetal bovine serum (FBS). (**B**) 10% FBS.

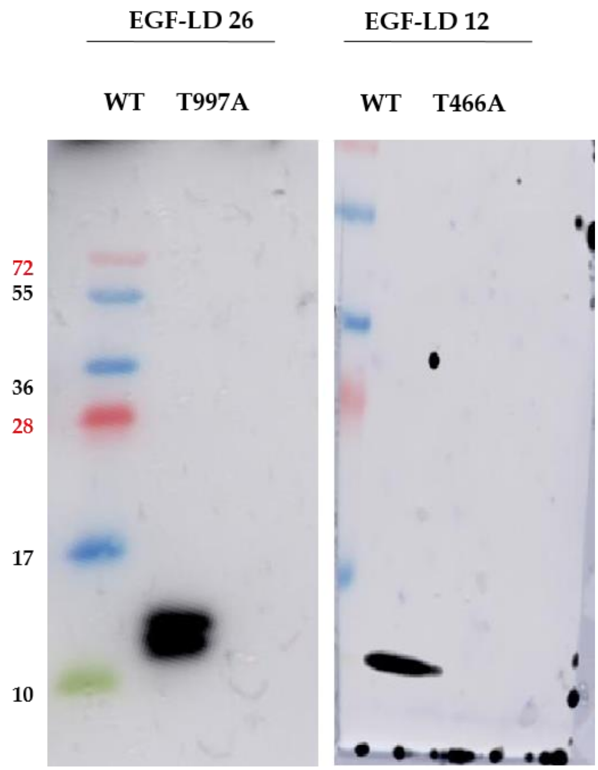

Figure S2. Uncropped blots of Figure 3a. In vitro *O*-fucosyltransferase assay for activity of WT and mutated POFUT1 variants using click chemistry.

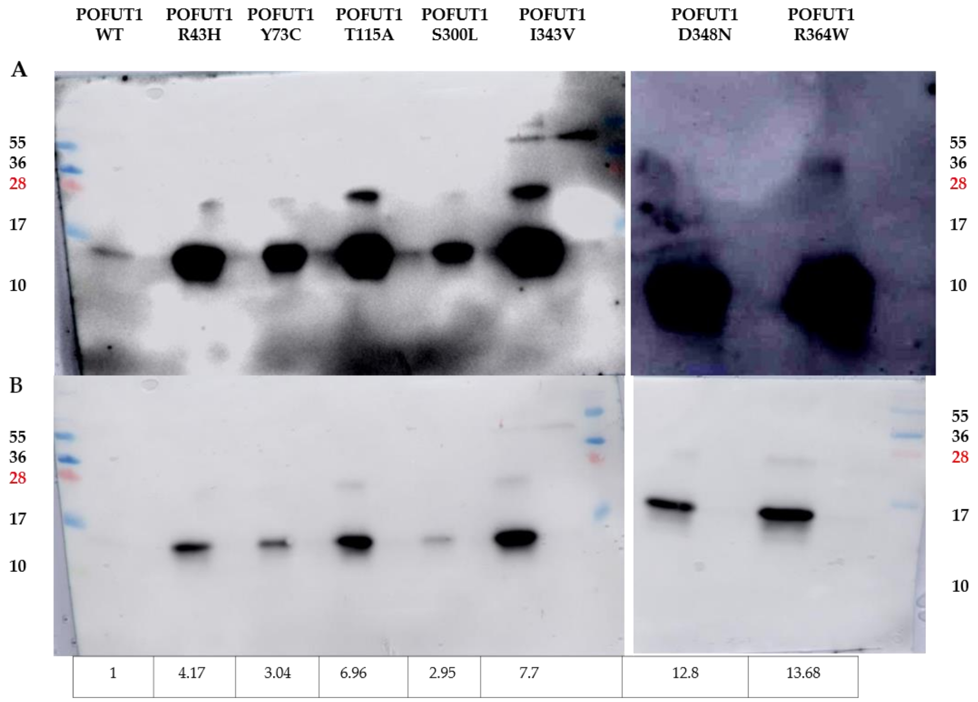

1	4.17	3.04	6.96	2.95	7.7	12.8	13.68

Figure S3. Uncropped blots of Figure 3b. In vitro *O*-fucosyltransferase assay for activity of WT and mutated POFUT1 variants using click chemistry under (**A**) high exposure. (**B**) low exposure.

© 2020 by the authors. Licensee MDPI, Basel, Switzerland. This article is an open access article distributed under the terms and conditions of the Creative Commons Attribution (CC BY) license (http://creativecommons.org/licenses/by/4.0/).
